# Structural Engineering in Piezoresistive Micropressure Sensors: A Focused Review

**DOI:** 10.3390/mi14081507

**Published:** 2023-07-27

**Authors:** Yan Liu, Xin Jiang, Haotian Yang, Hongbo Qin, Weidong Wang

**Affiliations:** 1 School of Mechano-Electronic Engineering, Xidian University, Xi’an 710071, China; 21041212042@stu.xidian.edu.cn (X.J.); 17040210008@stu.xidian.edu.cn (H.Y.); qhb0920qhb@xidian.edu.cn (H.Q.); 2 CityU-Xidian Joint Laboratory of Micro/Nano Manufacturing, Shenzhen 518057, China

**Keywords:** micropressure sensor, piezoresistive, structural engineering, diaphragm, piezoresistor

## Abstract

The longstanding demands for micropressure detection in commercial and industrial applications have led to the rapid development of relevant sensors. As a type of long-term favored device based on microelectromechanical system technology, the piezoresistive micropressure sensor has become a powerful measuring platform owing to its simple operational principle, favorable sensitivity and accuracy, mature fabrication, and low cost. Structural engineering in the sensing diaphragm and piezoresistor serves as a core issue in the construction of the micropressure sensor and undertakes the task of promoting the overall performance for the device. This paper focuses on the representative structural engineering in the development of the piezoresistive micropressure sensor, largely concerning the trade-off between measurement sensitivity and nonlinearity. Functional elements on the top and bottom layers of the diaphragm are summarized, and the influences of the shapes and arrangements of the piezoresistors are also discussed. The addition of new materials endows the research with possible solutions for applications in harsh environments. A prediction for future tends is presented, including emerging advances in materials science and micromachining techniques that will help the sensor become a stronger participant for the upcoming sensor epoch.

## 1. Introduction

Aligning with the rapid advancements in silicon micromachining techniques, microelectromechanical system (MEMS) sensors have enjoyed the highest sales in the sensor market. Piezoresistive sensors, basing their operating principle on the piezoresistivity of functional materials, are one of the MEMS devices first to be developed and have revealed great capacity in detecting acceleration, pressure, force, fluid, sound, strain, stress, etc. [1,2,3,4,5,6,7,8,9]. Piezoresistive micropressure sensors have great potential in many domains, such as tire pressure measurement systems for vehicles [10], biomedical catheters for healthcare [11,12], and pressure detectors for home appliances [13]; this can be attributed to their mature fabrication, structural simplicity, and excellent measuring performance [14]. The involved fluidic pressure basically falls in the range of thousands of Pascals. Meanwhile, scientific interest in the research on micropressure sensors is still experiencing stable and persistent effort, which is stimulated by constantly evolving application requirements and progress in material science and micromachining techniques.

Generally, there are two critical issues for the development of a piezoresistive micropressure sensor: the trade-off between sensitivity and linearity and the capacity for meeting the specific application requirements [15]. The first issue relates to the mechanical features of the sensing diaphragm [16]. The high sensitivity requires a large ratio of (membrane length)/(membrane thickness) to produce a more favorable sensing stress and then trigger a larger variation in the piezoresistors. Meanwhile, a large length/thickness ratio will cause a much swifter increase in the nonlinearity of pressure-to-stress conversion, appreciably impacting the device linearity. One solution for alleviating the restriction between sensitivity and nonlinearity is introducing engineered structures into the conventional flat. Many modified diaphragms have been explored to promote the comprehensive performance of piezoresistive micropressure sensors via using local stress concentration and partial stiffing components. Optimization of the shape and arrangement of piezoresistors also plays a role in performance enhancement. As for the second issue, the demands on device minimization and survivability in harsh environments necessitate more stringent requirements for research regarding piezoresistive micropressure sensors, and new forms of structural engineering based on the contributions of new materials have become a possible solution.

The continuous progress in design concept, material science, and micromachining techniques has allowed micropressure sensors to become an enduring field in MEMS sensors. Along with these emerging achievements, some review papers on this theme have been published. In 2014, Kumar et al. published an excellent review on the principles and considerations when designing an ‘ideal’ piezoresistive pressure sensor [17]. Besides the basic sensing principle and analytical expressions, a number of important factors that influence device performance were considered, and the tactics for improving sensing performance were summarized. Other reviews focus on the specializations of micropressure sensors, such as device minimization [18] and applications for biomedical catheters [11]. It is worth mentioning that the most recent scientific reviews discovered using the keyword ‘pressure sensor’ point to flexible pressure sensors, which mainly deal with contact force or tactile measuring. Nearly no attention is paid to recent efforts devoted to structural engineering for MEMS piezoresistive micropressure sensors. 

In this paper, the milestones and advances in structural engineering for MEMS piezoresistive micropressure sensors are reviewed, especially focusing on the modifications and optimizations in sensing diaphragms and piezoresistors. The roles of new materials are discussed, and future research trends are presented. Most of the cited literature is obtained by searching the database Web of Science using the keywords ‘piezoresistive micropressure sensor’ and the citation maps of many classic studies. The ones with the theme of flexible wearable pressure sensors are not the main target of this paper. This paper begins with a description of the basic elements of a piezoresistive micropressure sensor. Then, the structural engineering of the diaphragm and piezoresistor are presented in Section 3 and Section 4, respectively. Section 5 shows the contributions of new materials in promoting the structural engineering of pressure sensors. A prediction for future trends is given in Section 6, followed by a final conclusion.

## 2. Fundamentals for Piezoresistive Micropressure Sensors

**Figure 1:**
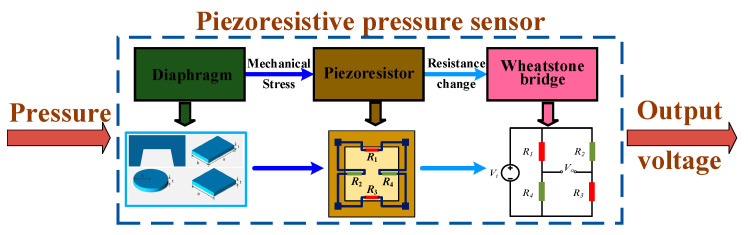
The basic components and principle for a piezoresistive micropressure sensor.

Piezoresistivity is a phenomenon in which the electrical resistance of a material changes in response to mechanical stress. As a kind of piezoresistive sensor, piezoresistive micropressure sensors also follow this mechanism; they encounter factors influencing piezoresistive performance and require a practical sensing structure to convert pressure into mechanical stress. Typically, a MEMS piezoresistive micropressure sensor consists of a sensing diaphragm for pressure capture and a group of piezoresistors in the form of a Wheatstone bridge circuit for signal transduction, as shown in Figure 1. This kind of device remains the basic operating principle just as with its inceptive approaches. Initially, the resistance of all piezoresistors was equal, and the Wheatstone bridge had no output voltage. When pressure is loaded, the diaphragm is deflected, and mechanical stresses are induced, which causes change in the resistance of piezoresistors. Then, the equilibrium state of the Wheatstone bridge is broken, and an output voltage can be measured, which usually has a tight correlation with the loaded pressure. If neglecting the possible deviation between the initial states of piezoresistors, the captured output voltage from the full bridge circuit can be written as

(1) $$V_{o}=\frac{1}{4}(\frac{\Delta R_{1}}{R_{1}}-\frac{\Delta R_{2}}{R_{2}}+\frac{\Delta R_{3}}{R_{3}}-\frac{\Delta R_{4}}{R_{4}})V_{i}$$

where Δ*R_i_* is the resistance change of each piezoresistor, Δ*R_i_*/*R_i_* is the relative variation, and *V_i_* is the supply voltage.

In order to take advantage of the conversion and compensation features of the Wheatstone bridge, the orientation of piezoresistors should be carefully determined to make sure that the resistance of two piezoresistors will increase and that of the other two will decrease when pressure is loaded; this is guaranteed by appropriately using the transverse and longitudinal stresses on each piezoresistor. Correspondingly, the resistance variation for the p-silicon piezoresistors aligned along [110] direction on (100) wafers can be calculated using [19]

(2) $$\frac{\Delta R_{i}}{R_{i}}=\frac{1}{2}\pi_{44}(\sigma_{li}-\sigma_{ti})$$

where *σ_li_* and *σ_ti_* are the longitudinal, transverse stresses sensed by the *i*th piezoresistor, and *π*_44_ is the piezoresistive coefficient. Usually, the stress difference (*σ_l_ − σ_t_*) of each piezoresistor is same in amplitude and may be opposite in sign. When triggered by a pressure load *P*, the measurement sensitivity *SEN* of the whole bridge circuit can be expressed as [20,21]

(3) $$SEN=\frac{1}{2}\pi_{44}(\sigma_{l}-\sigma_{t})V_{i}/P$$

It can be seen that the measurement sensitivity is strongly associated with the stress of piezoresistors, which can be regulated via the configuration of the diaphragm and the arrangement of the piezoresistors. As for the schemes with conventional flat diaphragms in Figure 1, circular-, square-, and rectangular-shaped diaphragms have been successively proposed. Circular and square diaphragms can generate a larger mechanical stress, leading to better sensitivity; rectangular diaphragms show a decreased tendency regarding the difference of transverse and longitudinal stresses when the length–width ratio of the diaphragm increases, which will influence the measurement sensitivity. Therefore, the former two diaphragms, especially the ones with the square scheme, are more popular in the construction of micropressure sensors. Moreover, sensitivity can also be promoted by optimizing the location and orientation of the piezoresistors, which collectively determine the stress difference of each piezoresistor.

Device nonlinearity error is a critical parameter for evaluating the accuracy of a sensor. Generally, the sensor accuracy *ACC* is characterized by three types of errors: hysteresis *HY*, repeatability *RE*, and nonlinearity *NL*. Then, the accuracy *ACC* is calculated as [22]

(4) $$ACC=\sqrt{HY^{2}+RE^{2}+NL^{2}}$$

In order to ensure measurement accuracy, it is necessary to effectively reduce the nonlinearity error (namely promote sensor linearity), which is defined as the maximum deviation of voltage between the calibration point and specified calibration curve. The value is often normalized via full-scale output voltage *V_o_*(*P_m_*), and its formula at a certain point can be written as [17]

(5) $$NL_{j}=\frac{V_{o}(P_{j})-\frac{V_{o}(P_{m})}{P_{m}}P_{j}}{V_{o}(P_{m})}\times 100\%$$

where *P_j_* is the pressure at calibrated points, *P_m_* is the maximum operation pressure, and *V_o_* is the corresponding voltage output. There are several sources for the nonlinearity of a micropressure sensor [23]. First, the nonlinear relationship between generated mechanical stresses and loaded pressure may derive from the balloon effect in large deflected diaphragms. When under a large fluidic pressure, the deflection of the central region will be obviously greater than that of the edge region due to supporting strength between the two regions. This deformation distribution is similar to an aerated balloon, and it will lead to nonlinearity in the pressure-to-stress conversion. Secondly, the nonlinearity of the piezoresistive effect may make the variation of resistance disproportionate to the stress when high mechanical stress is generated. The second issue can be conveniently reduced using an appropriate sensor design and fabrication. With a desired arrangement, the opposite behaviors of piezoresistors under tension or in compression can partially self-compensate the nonlinearity. However, the balloon effect in the micropressure sensor cannot be easily treated using common diaphragm designs. For the flat diaphragm with a length *L* and thickness *H*, the sensitivity is directly proportional to the ratio *(L/H)*, yet the nonlinearity of pressure-to-stress conversion is proportional to *(L/H)*^4^. This relationship means the nonlinearity has a faster increasing tendency when sensitivity is promoted, and there is an obvious constraint relationship between them [24]. The trade-off between sensitivity and linearity has become a critical issue in the development of high-performance piezoresistive micropressure sensors. In order to promote sensitivity, the diaphragm has to produce a larger deflection to enlarge the mechanical stress for piezoresistors; meanwhile, a stiffer diaphragm is often required to subduct the balloon effect and then limit the device’s nonlinearity. This dilemma cannot be solved by simply changing diaphragm size, and the assistance from structural engineering can help a lot.

## 3. Structural Engineering in Diaphragms

**Figure 2:**
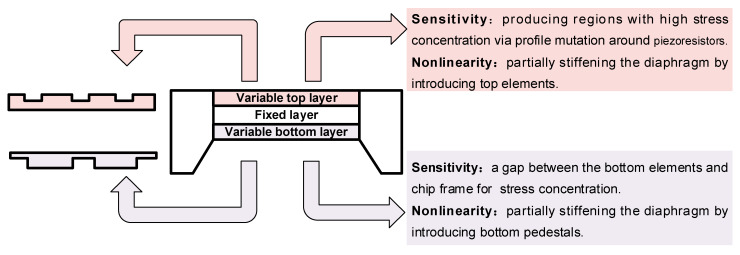
Possible considerations for structural engineering in sensing diaphragms.

Structural engineering in a diaphragm mainly reforms the geometry and cross profile of the diaphragm to realize the trade-off between sensitivity and linearity. Figure 2 shows the available considerations for structurally engineering the sensing diaphragm. This technique often uses the stress concentration (SC) effect to enlarge the mechanical stress in the piezoresistor region and guarantee device sensitivity; the introduction of partially stiffening elements gives the diaphragm a higher support strength to limit its maximum deflection within the range below one fifth of its thickness, which can alleviate the structural balloon effect and guarantee device linearity. Generally, the structural engineering in a diaphragm can be conducted by introducing new elements into two variable layers: the top layer and bottom layer. The structural engineering on the top layer can reform the shape of a diaphragm from the top surface by removing some elastic materials in the design domain. Then, the bottom layer often utilizes boss, peninsula, and rood beam to realize both SC and partial stiffening for the diaphragm. Their formation often correlates with the etching process of the back cavity. Several approaches may introduce engineered elements into both layers to provide a broader space for diaphragm design. Meanwhile, the reformation of the diaphragm greatly increases the complexity of the sensing structure, which also presents a great challenge in determining core dimensions. This section, regarding the two modified layers, will respectively review the reported works for the structural engineering within a diaphragm. Moreover, the efforts toward modeling and optimizing the structures are also discussed.

### 3.1. Structural Engineering on the Top Layer

Structural engineering on the top layer of diaphragm is realized via an additional etching from the top surface of the sensor chip, which processes the flat surface of the conventional C-type structure into multiple rugged areas with different functions, such as the SC element, partially stiffening element, and surface reforming region. Beam–membrane structure and groove structure are the two most popular approaches.

#### 3.1.1. Beam–Membrane Structure

**Figure 3:**
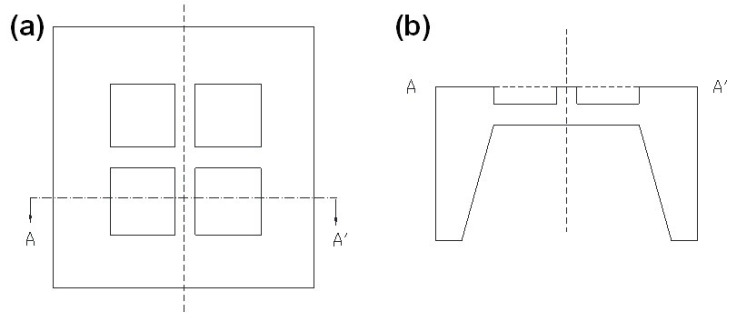
The diagram for CBM structure: (**a**) top view and (**b**) sectional view. Reproduced with permission from [26].

**Figure 4:**
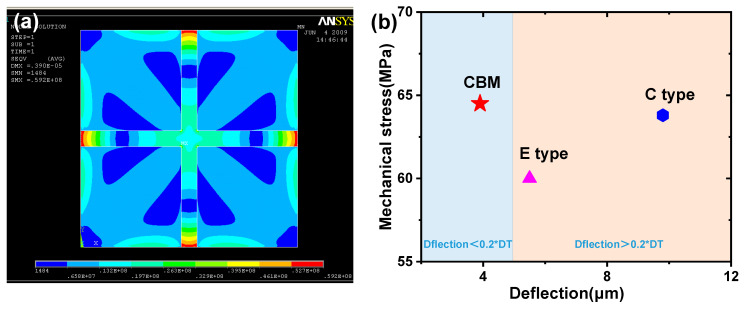
The mechanical feature of CBM structure: (**a**) stress distribution under 10 kPa pressure and (**b**) comparison of sensing stress and deflection between CBM, E-type, and C-type diaphragms. Reproduced with permission from [26].

Beam–membrane structure is a scheme that simultaneously concerns measurement sensitivity and diaphragm stiffness. It can be established via combining a C-type flat diaphragm and a partially stiffening beam. Figure 3 shows the micropressure sensor with a cross beam–membrane (CBM) structure [25,26]. In this scheme, the appearance of the cross beam obviously promotes the support strength of the whole structure and ensures a low level of structural balloon effect. Meanwhile, the locally protrusive state of the beam can produce an abrupt change in the cross-section area and concentrate the sensing stress to the ends of the beam, where the piezoresistor can obtain favorable stress (Figure 4a). Figure 4b indicates the maximum sensing stress and deflection of CBM, E-type, and C-type diaphragms under 10 kPa pressure. The area and thickness of each diaphragm is the same (2900 μm × 2900 μm for area and 20 μm for thickness); the cross beam has a width of 210 μm and a thickness of 20 μm; and the central boss in the E-type diaphragm has a dimension of 800 μm × 800 μm × 100 μm. The CBM scheme generates a minimum deflection of 3.9 μm, and the accompanying mechanical stress is slightly larger than that of other structures. Meanwhile, the deflections of E-type and C-type diaphragms all exceed the threshold of linear deformation (namely the 1/5 of diaphragm thickness). It can be seen that the CBM structure has a remarkable capacity in promoting the overall performance of the sensor, which can significantly diminish the device nonlinearity by reducing the diaphragm deflection and maintaining the sensing stress at a favorable level.

**Figure 5:**
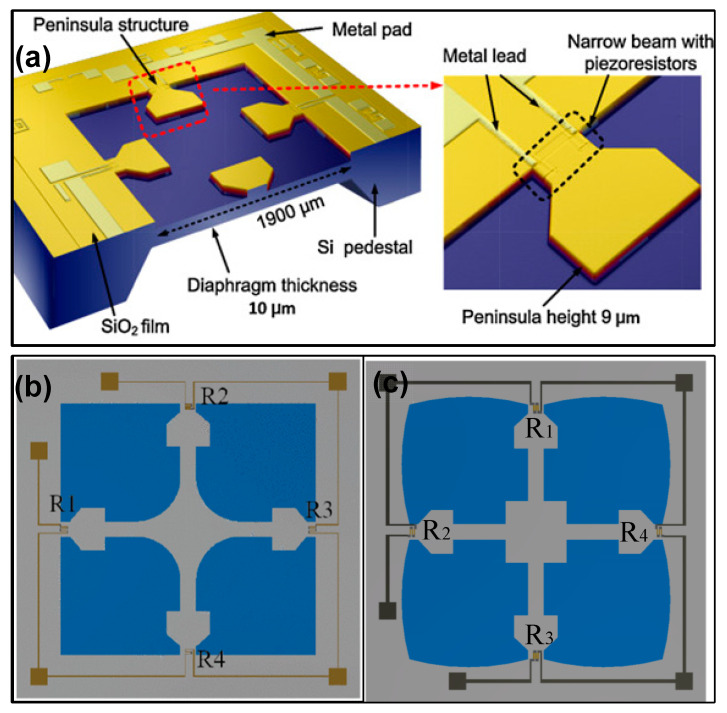
Three typical beam–island configurations for piezoresistive micropressure sensors: (**a**) peninsula structure, (**b**) cross beam–membrane and peninsula structure, and (**c**) improved structure with four petals. Reproduced with permission from [27] and under the terms and conditions of the Creative Commons Attribution license of [28,29].

In order to further leverage the advantages of the beam–membrane structure, many variations of this configuration have been sequentially developed. The configurations with differing beams and islands are becoming more and more abundant, and three typical beam–island configurations are illustrated in Figure 5.

The peninsula-structured diaphragm is a variant of the CBM scheme. Huang et al. introduced four peninsulas into the top layer of the diaphragm [27]. Compared with the CBM structure, the new configuration increases the maximum value of the difference in transverse and longitudinal stresses of 43.2%, and their nonlinearity error is very close. Further, Wu et al. presented an isosceles trapezoidal beam–membrane (ITBM) configuration on a square diaphragm [30]. Four isosceles trapezoidal beams (ITBs) were symmetrically settled on the edges of a square diaphragm, and the length of ITBs was 14.5% (580 μm/4000 μm) of the diaphragm. According to the simulated results, the decrease in junction width between ITBs and chip frame produces an increasing trend for sensitivity. Therefore, the ITBM scheme has the highest sensitivity compared to the conventional C-type and beam–membrane schemes. Although the deflection of each scheme is not discussed in the simulation, the obtained experimental results verified that the ITBM sensor has lower nonlinearity with respect to that of the other two schemes. These findings indicate that it is possible to balance the contradiction between sensitivity and nonlinearity by using the ITBM configuration. Tran et al. combined the cross beam–membrane and peninsula structure in a square diaphragm (named the CBMP structure), and a fillet island was settled in the center of diaphragm [28,31]. Compared to the C-type diaphragm, the CBMP sensor can achieve a 50% increase in sensitivity and a 32% decrease in maximum deflection. When compared to Tian’s CBM structure, a 16.4% increase for sensitivity and 12% decrease for maximum deflection can be achieved. Furthermore, the square diaphragm can be replaced by four-petal membranes to pursue better sensitivity [29]. The maximum longitudinal stress can be enlarged from 45.3 MPa to 63.0 MPa, and the accompanied variation of deflection can be limited by adding a back-center boss. A shuriken-shaped beam–membrane (SSBM) structure was proposed by Guan et al. to improve both sensitivity and linearity for the piezoresistive pressure sensor [32]. In this configuration, the junction width between the beam and chip frame is also reduced to enhance the SC effect. Simultaneously, the crossed shuriken beams obviously enlarge the central boss, which provides better local stiffness for the diaphragm and thus diminishes the deflection.

The following characteristics can be found in these mentioned structures. Firstly, a narrow joint is often settled between the beam and chip frame on the diaphragm. The SC effect can be achieved to guarantee the amplitude of mechanical stress, providing a favorable measurement sensitivity for the sensor. Meanwhile, the beam often has a wider end near the center of the diaphragm, and the boss structure is also popular, which can partially stiffen the central region of the diaphragm and then decrease the device nonlinearity via alleviating the structural balloon effect. In addition to achieving a good trade-off between sensitivity and nonlinearity, the SC effect induced via beam–membrane structure can give the small diaphragm a stress comparable to that of larger diaphragms. According to the results in Ref. [25], the CBM diaphragm only needs to occupy 21.6% of the area of C-type diaphragm to achieve competitive sensing stress. This superiority provides great potential for reducing chip size, improving integration level, and lowering device cost.

#### 3.1.2. Groove Structure

**Figure 6:**
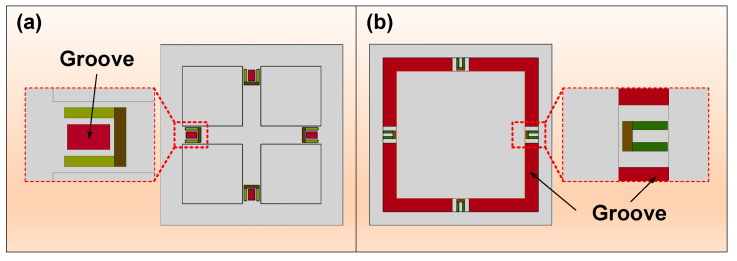
Illustration of groove structures: (**a**) local groove and (**b**) annular groove.

Groove structure is often achieved by locally thinning the stiff diaphragm to induce a sharp variation in structural cross profile [33], and the accompanying SC effect can lead to improvement of measurement sensitivity. As shown in Figure 6, there are two modes with which to introduce grooves: the local groove (LG) near the piezoresistor and the annular groove (AG) on the membrane. The former aims at directly enlarging the mechanical stress loaded to piezoresistors; the latter is intended to produce a SC effect similar to that of a beam–membrane structure. It is worth noting that the AG here is not intended to completely occupy all the boundaries of the diaphragm. The grooves directly etched on the surface of membrane fall into this category. Moreover, a decrease in diaphragm strength will occur during implement of grooves, causing a possible risk to device linearity. Thus, the grooves are often constructed in stronger diaphragms to guarantee the comprehensive performance of pressure sensors.

**Figure 7:**
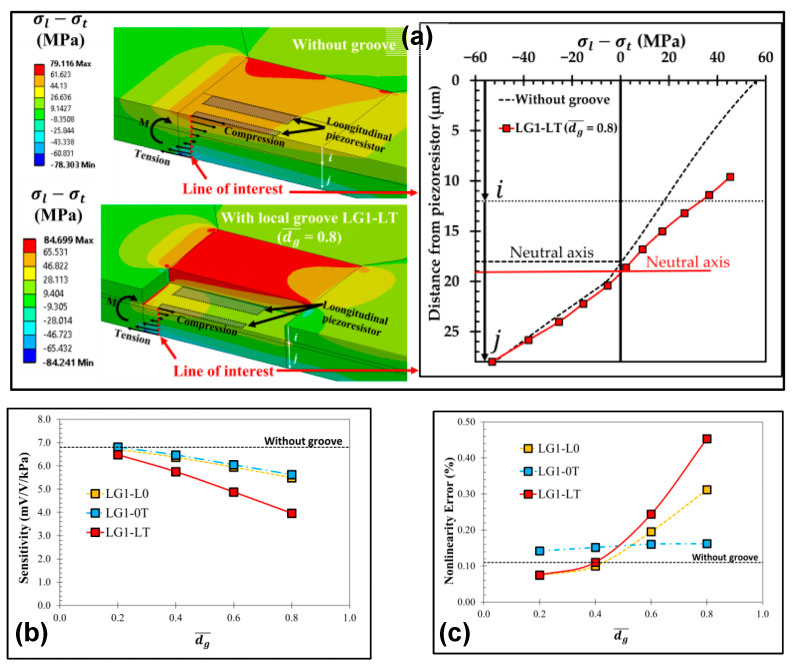
The effects of introducing LGs according to Thawornsathit et al.: (**a**) the effect on piezoresistor location and corresponding variations in stress; the effect on (**b**) sensitivity and (**c**) nonlinearity. Reproduced under the terms and conditions of the Creative Commons Attribution license of [34].

LGs are usually utilized in conjunction with beam–membrane structures, and they are settled around the piezoresistors to further increase the stress in piezoresistors’ locations. However, differences in modeling and treating methods lead to an obvious discrepancy in the function of introducing local grooves. As shown in Figure 7, Thawornsathit et al. constructed a model in which the piezoresistor plane went down with the appearance of LGs, and they investigated the influence of groove depth on device sensitivity and nonlinearity [34]. According to their results, the referred LG reduces the vertical distance between the piezoresistor and neutral plane and then diminish the effective stress. The stress of the piezoresistor decreases as the groove deepens, regardless of whether the grooves are located at longitudinal piezoresistors (𝐿𝐺1-𝐿0), transverse piezoresistors (𝐿𝐺1-0𝑇), or neither longitudinal nor transverse piezoresistors (𝐿𝐺1-𝐿𝑇). This is not desirable for the development of highly sensitive sensors. Meanwhile, device linearity also deteriorates along with the increase in groove depth. However, the mentioned piezoresistor arrangement with a lower surface is not a regular configuration for most piezoresistive pressure sensors, due to the difficulty in realizing piezoresistor and reliable ohmic connection. A more practical way for creating LGs is etching the grooves in the beam regions around piezoresistors without changing the resistor position. Gao et al. proposed a new peninsula structure diaphragm (NPSD) by combining Huang’s scheme [27], a cross beam-circular boss, and rectangular grooves at the root of peninsulas [35]. Though the cross beam-circular boss element further improves the diaphragm strength, the mechanical stress for piezoresistors increases because of the added rectangular LGs. Compared to the original peninsula device, the sensitivity of the NPSD sensor increases by 23.4%, while the nonlinearity is reduced by 69%. Obviously, LGs with proper arrangement are instrumental in promoting the comprehensive performance of piezoresistive pressure sensors.

**Figure 8:**
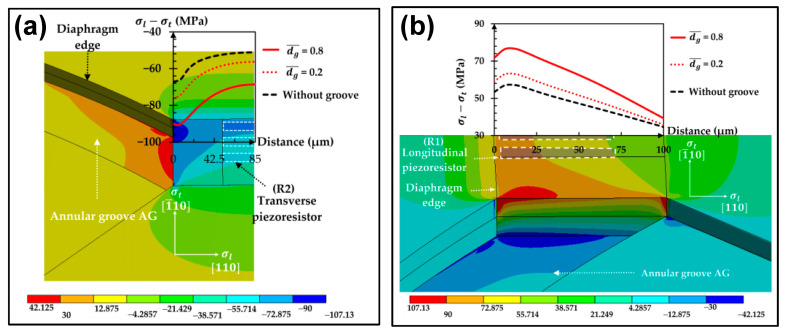
The effects of introducing AGs using the method of Thawornsathit et al.: (**a**) in the stress of the transverse piezoresistor and (**b**) in the stress of the longitudinal piezoresistor. Reproduced under the terms and conditions of the Creative Commons Attribution license of [34].

Annular grooves refer to the grooves on the surface of a diaphragm, and their task is to produce a SC effect similar to that of the beam–membrane structure or to further elevate the existing SC of beam–membrane structures. For example, Thawornsathit et al. also investigated the effectiveness of AGs (Figure 8) [34]. Compared with the original diaphragm, the AG-based structure can provide an increase of 14% to the sensitivity and a decrease of 35% to the nonlinearity. Zhang et al. validated the positive function of AGs via an annularly grooved conventional circle diaphragm, producing a sharp variation in the diaphragm cross section for SC [33]. Both FEM and experimental data proves that the benefit of groove structure is derived from forcing the stress to concentrate around the rib region, resulting in a 2.5 times enhancement to device sensitivity.

### 3.2. Structural Engineering on Both Layers

Along with continuous development of the design concept for piezoresistive pressure sensors, many researchers have also paid attention to engineering the bottom layer of the sensing diaphragm. Due to the fact that bottom structures do not conflict with top ones, the two structural groups can cooperate to greatly enrich the practical configurations.

#### 3.2.1. Available Elements for the Bottom Layer

The structures in the bottom layer are often manufactured to create a back cavity in the piezoresistive micropressure sensors. Limited by the micromachining technique, the thickness of bottom elements is much larger than that of top ones, leading to an obvious enhancement of the local stiffness of the diaphragm. Carefully optimizing the area and location of bottom elements is essential in structural engineering, avoiding excessive stiffness. Moreover, the bottom elements can act as displacement limiters under overloaded conditions. 

**Figure 9:**
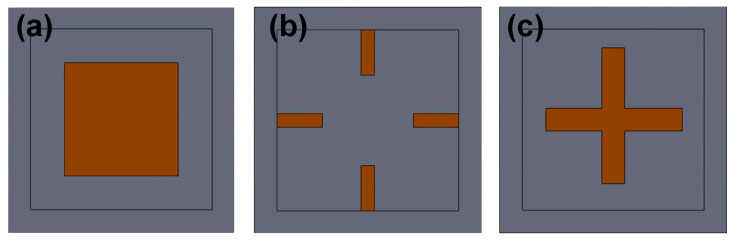
Common bottom elements for sensing diaphragms: (**a**) bottom boss, (**b**) bottom peninsula, and (**c**) bottom rood beam.

Figure 9 shows three common bottom elements for sensing diaphragms, including boss, peninsula, and rood beam [36,37,38]. The bottom boss plays a similar function to the top one, viz., locally stiffening the diaphragm. Its high thickness can turn the covered region into a rigid-like body without obvious deflection under a pressure load. The peninsula and rood beam can be divided into two cases: connected to the chip frame and not connected to the chip frame. In the former case, the stiffness of the diaphragm can be enhanced, and the sensitivity may suffer diminishment. In the latter case, the stiffening effect mainly occurs in the covered region and is not as strong as in the connected case. Meanwhile, the gap between peninsula/rood beam and frame can construct a new SC source. When this gap is aligned with the top piezoresistor region, the additional SC effect can further improve sensitivity. Therefore, many researchers choose the latter scheme when they introduce a peninsula/rood beam into the sensing diaphragms.

Theoretically, adding grooves to the bottom layer of a diaphragm can also increase sensor sensitivity. However, the large distance between the grooves and the bottom surface of the wafer makes it difficult to guarantee the accuracy of the dimensions using generalized micromachining techniques. Thus, back grooves are not widely applied in the creation of micropressure sensors.

#### 3.2.2. Configurations for Both-Layer Scheme

As previously mentioned, combining the top-layer element and bottom-layer element has become a favorable scheme for proposing high-performance piezoresistive micropressure sensors. Herein, the corresponding works are summarized in accordance with the utilized bottom-layer elements. 

**Figure 10:**
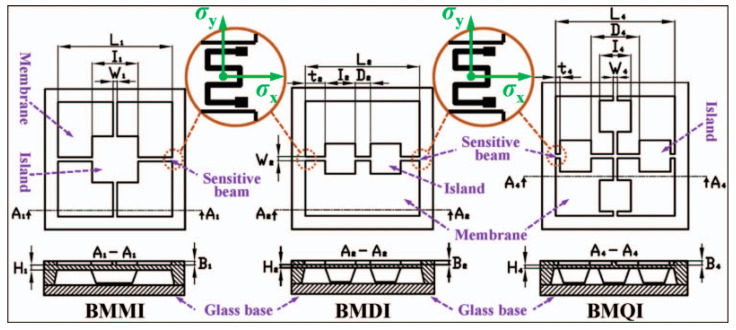
The schematic diagram of the front and cross-sectional views of BMMI, BMDI, and BMQI structures. Reproduced with permission from [44].

The scheme with top layer and bottom boss is a typical both-layer mode [39]. Yu et al. proposed a series of micropressure sensors via combining the top elements and bottom bosses [40,41,42,43]. In order to achieve the 500 Pa sensor for vacuum degree measurement, three beam–membrane–island geometries were settled on the top layer. In accordance with the number of utilized islands, these structures were named beam–membrane–mono-island (BMMI), beam–membrane–dual-island (BMDI), and beam–membrane–quad-island (BMQI), respectively (Figure 10). Meantime, same number of bottom bosses was located beneath the top islands, whose back surface was several microns lower than that of the wafer. The existence of these bottom bosses could locally strengthen the diaphragm and then alleviate the sensor nonlinearity. Characterizing results demonstrated that the BMQI sensor possessed the best sensitivity of 17.8 μV/(Pa·V), and the minimum nonlinearity was 0.2591%FS [41]. In addition, the motion of the bottom bosses would be limited by the bonded glass wafer when excessive pressure was loaded to the sensor chip, avoiding possible failure induced by overlarge deformation. By optimizing the gap between bottom bosses and glass wafer, these sensor prototypes all survived under a 200-times-overload condition.

However, the approaches with thick bottom bosses often suffer from acceleration-caused interferences [45]. Yu et al. also investigated this problem [44,46]. Concerning the convex loss of bottom boss during back wet etching, the responses of three devices to a 15 g acceleration were explored. The results showed that all approaches were sensitive to the loaded acceleration, and the maximum output voltage could reach 8.28% of the full pressure range value. Therefore, the thickness of the bottom boss should be carefully considered when using this scheme in the pressure measurement with vibration interferences. 

A bottom peninsula and rood beam are often paired with a top groove. Xu et al. combined the top groove with the bottom peninsula to achieve a 500 Pa pressure sensor with high sensitivity and low nonlinearity [47,48,49,50]. As mentioned above, the bottom peninsula did not connect with the chip frame, and an enhanced SC was created. The maximum sensing stress of the newly proposed diaphragm was increased by 375% compared with that of a conventional flat diaphragm. Meanwhile, the participation of the bottom peninsula decreased the nonlinearity from 0.8%FS to 0.23%FS. Similarly, Li et al. introduced rood beams into the grooved diaphragm to realize a high concentrated stress profile via aligning the SC regions generated by top grooves and a bottom rood beam, producing a local enhancement to the sensing stress [15,24,51]. Also, the bottom rood beam could partially stiffen the sensing diaphragm, limiting nonlinearity. The obtained prototype featured a sensitivity of 30.9 mV/V/psi and a nonlinearity of 0.21%FS, demonstrating the superiority of combining top and bottom elements. Different from the bottom boss with a large weight, a bottom peninsula and rood beam often take up a smaller area and therefore possess a smaller weight, which makes the acceleration interference no longer a serious problem.

### 3.3. Summaries for the Structural Engineering in Diaphragms

Many creative configurations have been proposed for structural engineering in sensing diaphragms, and a favorable trade-off between measurement sensitivity and nonlinearity has been achieved via different design concepts. Table 1 summarizes the performances of several sensors with representative diaphragms, concerning the diaphragm area, device sensitivity, and nonlinearity. In order to make the values more comparable, the sensitivity unit of each device is unified as mV/(V·kPa), and the unit for nonlinearity is set as %FS. 

The following factors can be found from the table above.

(a) Partial stiffening works well in improving device nonlinearity. Compared with the conventional sensor with a flat diaphragm, all the engineered structures achieve a distinct improvement in nonlinearity. The primary issue for constructing a partially stiffened diaphragm is maintaining the trade-off between sensitivity and nonlinearity, and it is not an easy task to outline a perfect guidance.

(b) The dimensions of a sensing diaphragm are closely correlated with the target measurement range. Though the introduction of structural engineering provides a possible solution for measuring the low pressure with a small diaphragm, a large dimension is still more favorable when testing ultralow pressures. The 500 Pa-ranged sensors with BMMI, BMQI, and BMDI diaphragms hold the largest chip area among the mentioned devices. Limited by the conventional etching process, the thickness of diaphragms cannot be guaranteed at the level of several microns, which also hinders the minimization of the sensing diaphragm. 

(c) The diaphragm with both-layer elements possesses certain advantages in improving device sensitivity, though additional dimensional parameters are involved. The burden from the fabrication of bottom elements is not a critical issue, due to its excellent compatibility with the process for the back cavity.

### 3.4. Modeling and Optimization of Diaphragms

With the continuous improvements in the structural engineering of micropressure sensors, the sensing diaphragm has become increasingly complex. This trend inevitably poses a great challenge for structural modeling. Multiple dimensional parameters are brought into the dominated group, and it becomes difficult to theoretically represent structural performance. Thus, several researchers analyze the correlation between sensor characteristics and structural dimensions using the finite element method (FEM) and curve fitting [25,29,41,42,43]. The effects of dimensional parameters in many configurations, such as the aforementioned CBM structure, BMMI/BMDI/BMQI structures, and CBMP structure, are presented in the described method. Firstly, the structural characteristics under a certain dimensional group are simulated, and the effecting tendency of each dimension can be obtained by continuously adjusting the size and repeating the simulation. Then, a power function is supposed to approximately correlate the performance of the proposed diaphragm (usually maximum deflection and maximum stress) and target dimensional parameters. Each unknown coefficient in the supposed function is sequentially determined using a curve fitting operation with the single variable method. For example, when the influence of diaphragm thickness *D* is studied, the value of *D* is changed in the range of the actual demand, and other variables are assumed to be constant. With the variation in *D*, a series of deflection and stress values are obtained from FEM. Based on these results, the approximate power functions that correlate deflection and stress with *D* can be derived from the curve fitting method, and the unknown coefficients can be determined. By repeating this process, several power functions for different dimensional parameters are derived. Then, an equation for modeling the whole diaphragm is established by combining all these power functions. 

The simulation-curve-fitting method provides an operational solution to modeling complex diaphragms, and the obtained function can be a reference for evaluating the performance’s dependence on each dimensional parameter. However, the heavy calculation load from the FEM simulation may become an obvious hindrance in the design and optimization of a sensing diaphragm. It will be challenging for researchers to rationally validate a large number of possible diaphragm configurations, due to time and resources consumed in numerically simulating all these designs. Meanwhile, the form of the supposed power function is chosen based on the experience of researchers, and the inaccuracy in curve fitting will decrease the presentation effectiveness of the obtained model. 

In order to accelerate the MEMS design cycle, some researchers turn to machine learning (ML) techniques for quick and accurate prediction of the physical properties of numerous geometric candidates without requiring massive FEM-based analysis [55]. Yigit et al. proposed an artificial neural network (ANN)-based model for MEMS diaphragm analysis [56]. A three-leaf clover (TLC) diaphragm was used to verify the effectiveness of this estimator, regarding static deflection and dynamic resonant frequency under different material and dimensional combinations. Firstly, FEM analysis data of 1680 different SiO_2_ TLC diaphragms were obtained to train, verify, and test the networks. Then, an ANN model considering different geometric and material parameters was established to quickly and accurately estimate the diaphragm responses without long design and simulation processes. Similarly, Guo et al. reported the use of deep learning techniques in calculating physical properties of MEMS structures [57]. In their approach, the geometries of candidate designs were represented by pixelated binary images, which were then labeled using numerical simulation results and used in the training of neural networks. After sufficient training and verification, the networks could calculate the physical properties of a vast number of candidate geometries and rank thousands of candidates in order to guide researchers toward good designs. Moreover, the calculation speed of the proposed networks is 2.6 × 10^4^ times faster than that of conventional numerical simulation packages with good accuracies of 96.8 ± 3.1% when modeling the quality factor of a disk-shaped microscale resonator.

It can be seen from the published work that the ML methods for investigating the responding patterns of different MEMS configurations have provided practical support for rapid iterations of design. However, the devices referred to in previous work often have a simple configuration, whose dimensional parameters are much less than the abovementioned sensing diaphragms. This distinction raises a doubt about the possibility of using ML methods in micropressure sensors. Moreover, the construction of neural networks still needs a great deal of FEM data for training, and the generalization ability of obtained ML models is also a critical issue when determining their migration capability. 

## 4. Structural Engineering in Piezoresistors

### 4.1. Arrangement of Piezoresistors

**Figure 11:**
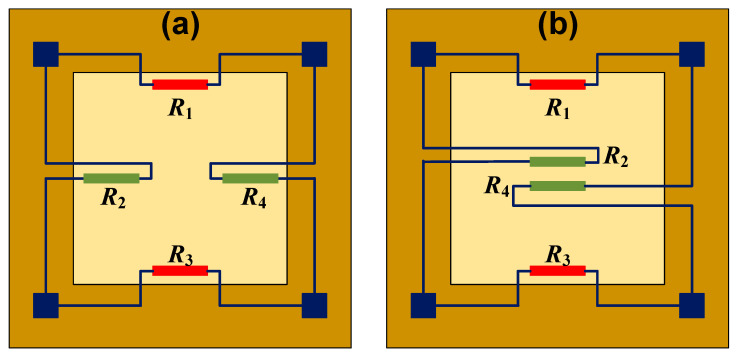
The common positions for piezoresistors in a square diaphragm: (**a**) all at the edge and (**b**) two at the edge and the other two at the center.

**Figure 12:**
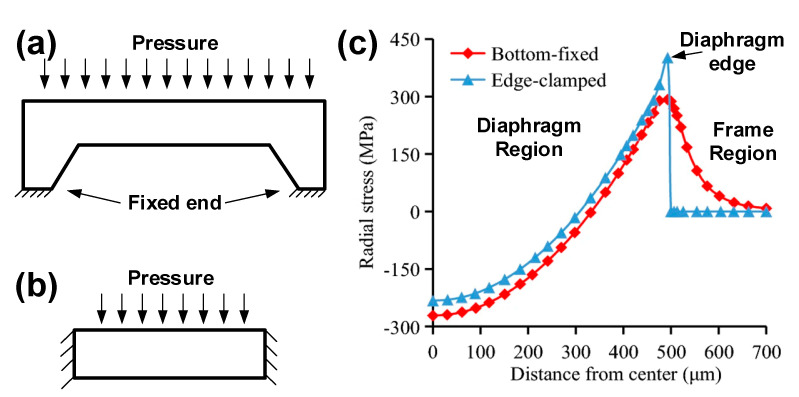
The working state of a diaphragm: (**a**) bottom fixed, (**b**) simplified edge clamped, and (**c**) the stress under different working states. Reproduced with permission from [61].

As mentioned above, there are usually four piezoresistors in the sensing diaphragm in order to construct a full Wheatstone bridge circuit. The piezoresistors are grouped in pairs: the variation tendency of resistance is same within the pair and is inverse between pairs. Thus, the settled directions of the piezoresistors should be carefully determined, concerning the stress distribution of diaphragm. Taking the flat square diaphragm for instance, the common positions for piezoresistors are shown in Figure 11. Firstly, all piezoresistors can be located at the edge of diaphragm, and two work as the transverse unit to sense the stress along their length, and the other two work as the longitudinal unit to sense the stress along their width. The transverse and longitudinal units will be stimulated by the stress with different positive/negative states, and they will produce a resistance variation with a different tendency. Then, the scheme in Figure 11b sets two piezoresistors at the center while setting the other two at the edge of the sensing diaphragm. The edge ones sense the tensile stress, and the two central ones sense the compressive resistance, and the resistance variation is also in accord with the requirement from the Wheatstone bridge. Generally, the former configuration is more popular in the development of pressure sensors, but the latter is also favorable in some special-shaped diaphragms [58,59,60]. As for the location of edge piezoresistors, some researchers have claimed that it is helpful in improving sensitivity to extend them beyond the diaphragm to the chip frame [36,61]. For most piezoresistive pressure sensors, the sensing diaphragm works under the bottom-fixed constraint shown in Figure 12a. Different from the simplified edge-clamped constraint (Figure 12b), the mechanical stress will distribute beyond the diaphragm’s edge, and it will generate a high-stress region within a certain area in the chip frame (Figure 12c). However, it is not a simple task to calculate the actual area for this region, making this method unpopular in the development of pressure sensors. 

In addition, some devices will set dummy piezoresistors in the nondeformable regions of a sensor chip to reduce the impact of temperature [62,63]. The dummies only respond to temperature variation and are not sensitive to measured pressure. Therefore, an output without temperature disturbance can be obtained by subtracting the responses of the dummies from those of sensitive ones.

### 4.2. Shape of Piezoresistors

**Figure 13:**
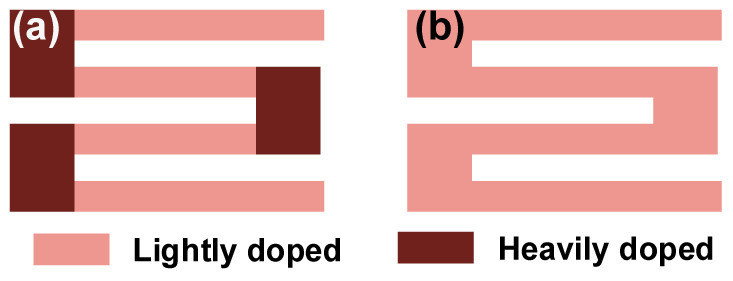
Shapes for piezoresistor: (**a**) with and (**b**) without heavily doped connecting regions.

As shown in Figure 13a, the piezoresistors in a micropressure sensor are often in a serpentine shape with a lightly doped piezoresistive region (LDPR) and heavily doped connecting region (HDCR). The LDPR possesses a larger piezoresistive coefficient, and it undertakes the task of transducing the mechanical stress of a diaphragm into resistance variation. The LDCR contains multiple folds to simultaneously guarantee favorable resistance and stress level for whole piezoresistors. HDCRs work as the connecting arms at the ends of each LDCR fold to reduce the transverse negative piezoresistive effect. In most of the published literature, only the LDPR is considered during device development. However, the HDCR also has resistance and a piezoresistive effect, and it will affect the sensor performances. The existence of a HDCR would reduce the sensitivity of a pressure sensor, and the reduction is proportional to the ratio of its resistance to the total resistance of the piezoresistor. Three different prototypes were manufactured and tested by Li et al. to highlight the impact of HDCRs, in which the length of the HDCR was significantly larger than that of the LDPR [64]. The experimental results showed that an 18% decrease in sensitivity would happen if the form of the HDCR was not designed correctly. Therefore, the dimensions of a HDCR should be carefully considered to reduce its resistance and then limit its influence on sensitivity. The existence of a HDCR also impacts the device linearity, and the piezoresistor without a HDCR (Figure 13b) can be a probable solution [65]. The tested results from prototypes with or without HDCRs demonstrated that the pressure sensors with S-shaped piezoresistors featured a 30% better linearity than those of the sensors with tri-meander-shaped piezoresistors. Meanwhile, sensitivity was reduced by about 15%, due to the loss of capacity in disposing of the negative piezoresistive effect of the connecting arm. In addition to paying attention to the design of a LDCR, the doping levels of the connecting arm regions should also be carefully considered according to the actual design target. If sensitivity is the primary goal, a HDCR should be used, and its resistance should be minimized as much as possible. When targeting linearity, it is possible to use the S-shaped piezoresistors to further improve this parameter. 

**Figure 14:**
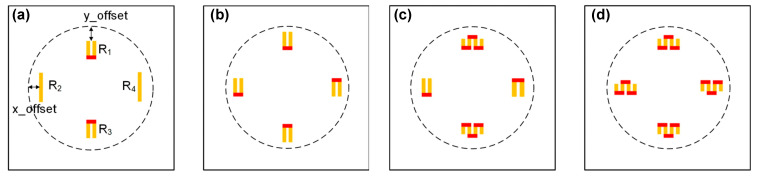
The models for simulating the influence of shape of LDCRs: (**a**) line-shaped and U-shaped; (**b**) all U-shaped; (**c**) U-shaped and W-shaped; (**d**) all W-shaped. Reproduced with permission from [61].

Further, according to the simulation in [61], the folding number of the LDCR also influences the device performance. The length of the LDCR remains unchanged, and four different models with line-shaped, U-shaped, and W-shaped piezoresistors are simulated (Figure 14). The results showed that the W-shaped piezoresistor is more suitable to sense transverse stress, and the line piezoresistor is better for longitudinal stress. The transverse stress is distributed along the edge of the diaphragm with a favorable width, and the piezoresistors with multiple folds can better take advantage of the width of this high-stress region; the transverse stress is in a strip-shaped form, and the piezoresistors with few folds can work better.

## 5. Contributions of New Materials 

### 5.1. New Materials for Sensing Diaphragms

Along with using new materials in the development of MEMS sensors, sensing diaphragms based on other materials are also built to fulfill emerging requirements for urgent applications, such as silicon carbide (SiC) for ultrahigh temperature sensors, advanced carbons for ultra-thin diaphragms, and polymers for new technology verification [66,67,68,69,70,71,72,73,74]. 

Though silicon-based pressure sensors have dominated the market due to silicon’s wide usability, mature surface, and bulk micromachining, the degradation of their mechanical properties and their piezoresistive effect at high temperature still limits their applications in geothermal measurement, gas turbines, and deep drilling. The SiC-based sensing diaphragm greatly extends the operating temperature from 400 °C to 800 °C, making it a superior candidate for the pressure sensor at elevated temperatures. As one of the third-generation semiconductor materials, SiC has a large energy band gap of 2.3 to 3.4 eV, superior mechanical properties (300–500 GPa for Young’s modulus and 9.15 for hardness), and excellent chemical inertness. Among the about 250 crystal types of SiC, the most attainable polytypes include the cubic crystal structure of 3C-SiC (β-SiC) and the hexagonal close-packed structure of 6H-/4H-SiC (α-SiC). The 3C-SiC can only heteroepitaxially grow on other materials (such as Si) in the form of thin films, which increases the difficulty of building all-3C-SiC devices and imposes an extra limitation on the operating temperature. In contrast, 6H-SiC and 4H-SiC can be epitaxially grown to produce commercial wafers for different all-SiC devices, and relevant pressure sensors have attracted significant attention. Wang et al. reported a serial of all-4H-SiC pressure sensors with a measurement range of 5MPa, in which the sensing diaphragm was produced via laser micromachining of the 4H-SiC wafer [75,76,77]. The experimental results showed that a low hysteresis error of 0.17%FS and nonlinearity of 0.20%FS could be achieved at room temperature. Meanwhile, the decrease in sensitivity with rising temperature is clearly exhibited: the sensitivity was 1.63 mV/V/MPa at −50 °C, 1.42 mV/V/MPa at 25 °C, and 1.04 mV/V/MPa at 300 °C. The decrease in sensitivity was attributed to the degradation of the piezoresistivity of the 4H-SiC at higher temperature. Shang et al. produced an n-type 4H-SiC absolute pressure sensor with a measurement range of 10 MPa [78]. The diaphragm with a 1000 µm diameter and 45 µm thickness was made by grinding and chemical mechanical polishing processes and then was tightly bonded with a SiC shallow groove structure. According to characterization results, hysteresis of 0.171%FS, repeatability of 0.232%FS, and a nonlinearity error of 0.034%FS were obtained. However, a drop of 50.7% in sensitivity also happened when the operating temperature rose from 30 °C to 200 °C.

**Figure 15:**
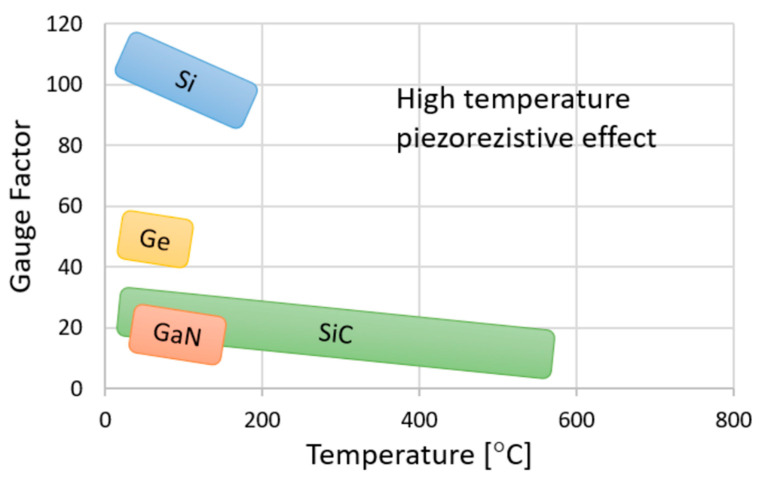
Gauge factor for typical semiconducting materials under different temperatures. Reproduced under the terms and conditions of the Creative Commons Attribution license of [79].

Concerning the features of SiC, there are two critical issues if applying it in micropressure sensors. Firstly, relatively low piezoresistivity and great temperature dependence makes it difficult to guarantee the sensitivity and temperature stability [66]. As shown in Figure 15 [79], the gauge factor (GF) of SiC is about 30 at room temperature, while the value of bulk Si is about 110. Then, the GF of SiC decreases by approximately 35% when the temperature increases from room temperature to 200 °C, which is also larger than Si. Furthermore, the Young’s modulus of SiC is about three times larger than Si, giving the diaphragm a much larger stiffness. Moreover, the lack of a high-efficiency bulk-micromachining method, induced via covalent bonds between Si and C atoms, greatly increases the difficulty of producing a thin sensing diaphragm. Obviously, the thick, stiff diaphragm is more suitable for high-pressure sensors, and thus the most-reported SiC pressure sensors range at the level of MPa. 

Reducing the size of a sensor chip has become another irresistible trend in the development of micropressure sensors, which requires a continuous reduction in diaphragm thickness to maintain sensitivity. The deep reactive ion etching and epitaxy process for bulk Si is becoming useless in fabricating ultra-thin (less than 1 μm) diaphragms. Therefore, exploration of using advanced carbons as structural materials has been conducted for micropressure sensors [80]. Li et al. reported a sensing diaphragm based on graphene–boron nitride heterostructure [81]. The five-layered diaphragm had a thickness around 200 nm and was placed onto a 17 μm × 6 μm rectangular cavity to build a pressure-sensing unit, realizing a sensitivity of 186.4 μV/(V·kPa). A procedure for fabricating ultra-thin nanocrystalline diamond membranes on glass was presented by Janssens et al. [82]. A 150 nm-thick circular diaphragm with a diameter of 555 μm can successfully sense the positive 0–0.7 bar differential pressure with a sensitivity of 0.6%/bar. The utilization of graphene and diamond-based ultra-thin diaphragms ensures favorable sensitivity for the minimized sensors. Nevertheless, their extremely high cost and difficulties in mass fabrication are still a great hindrance. To overcome this issue, researchers have turned to the diamond-like carbon (DLC) films, which feature superior physiochemical properties, high compatibility with MEMS micromachining processes, wide range of thickness (from monolayer to over 50 µm), and a unique GF up to 1200 [71]. Inspired by the aforementioned advantages, Ma et al. proposed a micropressure sensor with a self-supported 785 nm-thick DLC/Si_3_N_4_/SiO_2_ diaphragm [83]. Benefiting from its simple and economical manufacturing process, the production of the DLC-based micropressure sensor showed strong competitiveness in industrial fields. 

More recently, some interesting exploratory studies have been conducted to verify the possibility of further extending the scale of diaphragm materials. Polymers have been widely used in developing flexible force/tactile detectors in biomedical, healthcare, or aerospace applications, but very few studies refer to fluidic environment applications [84]. A new pressure sensor for applications in gaseous environments was reported by Balderrama et al.; their study’s novelty was the 130 µm-thick flexible polyethylene terephthalate (PET) diaphragm and a number of deposited nichrome piezoresistors [85]. The technology of using flexible materials in pressure sensors opened new opportunities for merging different materials in sensing diaphragms, but linearity and operable temperature are still far from desirable due to the characteristics of PET films. The other way for polymers to be used in pressure sensors is cooperating with 3D printing techniques. Liu et al. printed a micropressure sensor via digital light processing (DLP) and screen-printing techniques [86]. The transparent high-temperature resin from Formlabs was solidified by the DLP-based printer to form the sensor substrate, and the piezoresistors were made by screen printing a carbon paste. A GF of 17.01 ± 1.85 was obtained for the piezoresistors, along with a sensitivity of 0.91 mV/(V·kPa), and a nonlinearity of 2.077% FS was obtained for the sensor within the range 0~2.4 kPa. Meanwhile, some studies also validate the possibility of using a glass membrane as the sensing diaphragm for pressure sensors, and acceptable performance can be obtained [87,88]. 

### 5.2. New Materials for Piezoresistors

With the advances in material science and micromachining techniques, the available candidates for piezoresistors also obtain an expansion. The materials with desirable piezoresistivity and micromachinability will possibly become piezoresistors in pressure sensors. Firstly, similar to bulk Si, many structural materials for diaphragms also possess piezoresistivity, and the GF can be further promoted via proper doping. The abovementioned SiC, DLC, graphene, and diamond simultaneously adopt structural and piezoresistive roles. Then, the allotropes of carbon, such as carbon nanotubes (CNTs) and graphene, can be deposited onto the predetermined regions to produce desirable piezoresistors. Zhu et al. transferred a single-layer chemical vapor deposition graphene to the edge of a Si-sensing diaphragm to realize a high-repeatability graphene pressure sensor [89]. With protection from a Si_3_N_4_ nanofilm, the obtained device achieved a sensitivity of 5.51 × 10^−5^/kPa, repeatability error of 4.062%FS, and hysteresis of 2.118%FS, which is better than many typical graphene pressure sensors. Finally, attempts based on metal piezoresistors are also ongoing. Pt strain gauge [87], nichrome alloy (Ni_80_Cr_20_) [85], and silver nanoparticle array [90] have proven their piezoresistivity. However, the GF of metal gauges is not as excellent as other materials. 

### 5.3. Discussion on the Contributions of Flexible Conductive Materials

As described above, the organic, flexible, conductive materials for contact pressure and strain sensing also possess promising potential for fluidic measurement. These functional materials can capture the external stimuli and respond to them with resistance variation. Namely, they can simultaneously undertake the sensing task of the diaphragm and the conversion task of the piezoresistor. Different from the piezoresistive effect of silicon, the piezoresistive mechanisms of flexible conductive materials mainly include disconnection of conductive paths, crack propagation, and tunneling effect [91]. Generally, there are two kinds of composition in conductive materials: the elastomer as substrate and electrical conductivity as ameliorant [92,93]. Electrical conductivity must be present in the elastomer to achieve piezoresistive properties. An elastomer, such as rubber, plastic, or cellulose, is generally insulating, and conductive fillers or polymers can impart conductivity to an elastomer. The use of conductive fillers is a more promising strategy because such fillers can be used to control electrical conductivity and mechanical strength. Common conductive fillers include metal nanowires/nanoparticles, carbon allotropes (e.g., CNTs, graphene, and black carbon), and new 2D materials [94,95,96,97,98,99,100,101]. These fillers may be used alone or mixed. More recently, some efforts have been devoted to the exploration of hydrogels in piezoresistive devices, which can endow sensors with excellent adhesion and biocompatibility [102,103,104,105,106].

However, the use of organic flexible materials in high-performance piezoresistive micropressure sensors is still in its early stages, and there are several stumbling blocks in the path. Table 2 offers a simple qualitative comparison between the main properties of inorganic semiconductors and organic composites. Concerning the referenced parameters, the following issues may need to be further addressed. The first issue is linearity. Though the stretchability of organic materials gives a high operation strain to the flexible sensors, the accompanying too-large deflection also causes a nonlinear response for the sensors. The obtained nonlinearity error is much larger than that of silicon-based pressure sensors. This deficiency greatly limits the application of flexible sensors in a high-precision measuring system. Secondly, the allowable temperature of flexible sensors prevents them from operating stably for a long time at high temperatures, which is also not desirable in practical applications. Additionally, the dynamic response capability is also critical. Micropressure testing requires an operating frequency at tens of kHz to capture the rapidly changing pressures. Meanwhile, flexible devices often work with a response/recover time of milliseconds, which is too slow for the measurement of fluidic pressure and can be attributed to the low modulus of flexible materials. Obviously, flexible conductive materials have achieved great successes in flexible/stretchable pressure sensors and feature potential application in piezoresistive micropressure sensors, but a great revolution in material science, micromachining techniques, and design concepts is still needed to render the relevant devices with a measuring capacity similar to conventional silicon ones.

## 6. Future Trends

The performance of piezoresistive micropressure sensors has clearly been promoted via the elaborate structural engineering in sensing diaphragms and piezoresistors. Progress in high-performance materials constantly brings revolutionary solutions to this research. Meanwhile, current research is still far from perfection, and several directions for further research and development are attractive and flourishing:

(1) Intelligent generation for the structures: By now, most of the reported schemes for sensing diaphragms and piezoresistors originate from empirical accumulation and iterative optimization. Great time and effort are taken up by the long-term process of preliminary design, verification, modification, and reverification. Effectiveness is often dominated by the awareness and innovation capabilities of engineers. Currently, the application of artificial intelligence generated content (AIGC) has stimulated a huge storm in multiple industries [107]. Similarly, an intelligent generator for automatically and efficiently proposing sensing diaphragms and piezoresistors will be an unprecedented breakthrough in the development of piezoresistive micropressure sensors. Researchers may only need to accurately describe the required functionality, and then a complete structural solution could be generated. However, conventional research based on FEM simulation and curve fitting cannot sufficiently meet data volume requirements. The reported deep learning method for MEMS structural design is just in its infant stage, and only few simple non-parameterized geometries have been modeled based on this regulation. With the continuous accumulation of structural models, design criteria, and AI algorithms, the model library and tools can provide promising support for intelligent generators in the future. 

(2) Device minimization: Minimization has become an inevitable trend in the development of micropressure sensors, which creates new demands for the configurations of diaphragms and piezoresistors. The decrease of the diaphragm area requires a continuously reduced thickness to maintain device sensitivity. The applications of new micromachining techniques can be of great assistance. For example, a novel scar-free micro-hole inter-etching and sealing process can produce a tiny 0.4 mm × 0.4 mm piezoresistive absolute pressure sensor with a fabrication cost as low as 0.01 US$/die and a high throughput of 90,000 dies per 6-inch wafer [60]. Benefiting from the 8.5 μm-thick beam–island diaphragm, the obtained device features a measurement sensitivity of 0.27 mV/(V·kPa) and a nonlinearity of 0.10%FS. Furthermore, the stimulation from new materials also can promote device minimization, which has been discussed in the former subsection. However, many attempts at developing new micromachining and materials are still in the laboratorial stage, which acts based on a series of sophisticated instruments and complicated processes without considering cost, yield, and device reliability. With the achievements in wafer yield, lowered cost, and processing compatibility, applications of new micromachining techniques and materials will greatly accelerate device miniaturization in the near future.

(3) Device flexibility: A flexible resistance pressure sensor has been widely investigated as the force/tactile detectors for biomedical, automotive, or aerospace applications, but problems in nonlinearity, hysteresis, creepage, and tardy response make them unpopular for long-term stable measurements [92,108]. Thus, flexible fluidic pressure sensors for high-precision measurement systems have not been well studied. Generally, there are two possible ways to make the piezoresistive micropressure sensor flexible: developing a fully flexible chip and implementing hybrid integration. Developing a fully flexible micropressure sensor relies on great breakthroughs in functional materials and fabrication to overcome existing problems. The design procedures may also need an upgrade to adapt to the regulation of flexible electronics, and the achievements may become a new milestone for MEMS pressure sensors. Hybrid integration is expedient for realizing device flexibility, in which the MEMS sensor can be integrated onto the flexible substrate to realize macro flexibility. For example, a hybrid sensor system for concurrently monitoring heart electrical and mechanical functions was reported [109]. A solid accelerometer is adhered onto the polymer substrate, and the stretchable serpentine metal wires on the substrate guarantee electrical connection when the whole system is compliantly attached onto the largely curved surface for specific measuring tasks. Obviously, the latter scheme is more feasible under existing conditions, and a better minimization of sensor chips can better facilitate this kind of flexibility.

## 7. Conclusions

Structural engineering in sensing diaphragms and piezoresistors has endowed piezoresistive micropressure sensors with excellent sensitivity and linearity, and this can be further strengthened via utilization of new functional materials. As for the sensing diaphragm, many geometries, such as cross beam, groove, peninsula, island, and boss, have been introduced into the top and bottom layers of the diaphragm to produce stress concentration in the region of the piezoresistor to enhance measurement sensitivity. Meanwhile, partial stiffness of the diaphragm can also be achieved by adding these elements to reduce device nonlinearity. Shape and arrangement of piezoresistors are additional issues for adjusting sensor performance. The introduction of new materials can give the sensor extended features of ultrahigh operable temperature, thin diaphragm for minimization, and more efficient transducing ability. Though there have been great achievements in the development of piezoresistive micropressure sensors, great efforts are still needed to address issues in intelligent structural generation, device minimization, and flexibility. With ongoing progress in materials science, micromachining techniques, and intelligence engineering, piezoresistive micropressure sensors will overcome these hindrances and become an excellent candidate for the future sensor era.

## Figures and Tables

**Table 1:** Summaries of structural engineering in diaphragms.

Type	DC	DA (μm^2^)	Range (kPa)	SEN (mV/(V·kPa))	NL (/%FS)	Ref.
Null	Flat	1150 × 1150	100	0.23	0.58	[52]
Only top layer	CBM	2900 × 2900	10	1.61	0.19	[25]
	Peninsula-structured	1900 × 1900	5	3.68	0.36	[27]
	Shuriken-structured	1900 × 1900	3	4.72	0.18	[32]
	Modified beam–island	950 μm ^a^	1.2	3.654	0.05	[53]
	CBMP	2900 × 2900	5	5.16	0.28	[31]
	ITBM	4000 × 4000	3	1.928	0.09	[30]
	NPSD with groove	1900 × 1900	5	4.54	0.11	[54]
Both top and bottom layers	Top groove and bottom peninsula	3500 × 3500	0.5	65	0.33	[47]
	BMMI	5600 × 5600	0.5	11.1	0.196	[43]
	BMQI	5600 × 5600	0.5	17.8	0.14	[41]
	BMDI	5600 × 5600	0.5	16.1	0.259	[42]
	Top groove and bottom rood beam	3600 × 3600	6.89	4.48	0.25	[24]
	CBMP+ petal	3100 × 3100	5	6.934	0.23 ^b^	[29]

^a^ is the circum-circle diameter of the hexagonal-shaped diaphragm; ^b^ only simulation value. DC = Diaphragm configuration, DA = Diaphragm area, SEN = Sensitivity, NL = Nonlinearity.

**Table 2:** Qualitative comparison between the main properties of inorganic semiconductors and organic composites.

Property	Inorganic Semiconductor	Organic Composite	Relevant Sensing Parameter
Modulus	Low	High	Operating frequency
Allowable Temperature	Lower than 100 °C	Higher than 200 °C	Operating temperature
Durability	Medium	High	Performance stability under different conditions
Long-term stability	Medium	High	Longstanding stability for measuring capacity
Hysteresis	High	Low	Measurement accuracy
Creepage	High	Low	Measurement accuracy
Operation strain	High	Very low	Stretchability and linearity

## Data Availability

Not applicable.
